# Inhibition of mitochondrial LonP1 protease by allosteric blockade of ATP binding and hydrolysis *via* CDDO and its derivatives

**DOI:** 10.1016/j.jbc.2022.101719

**Published:** 2022-02-11

**Authors:** Jae Lee, Ashutosh K. Pandey, Sundararajan Venkatesh, Jayapalraja Thilagavathi, Tadashi Honda, Kamal Singh, Carolyn K. Suzuki

**Affiliations:** 1 Department of Microbiology, Biochemistry and Molecular Genetics, Rutgers - New Jersey Medical School, Newark, New Jersey, USA; 2 Department of Chemistry and Institution of Chemical Biology & Drug Discovery, Stony Brook University, Stony Brook, New York, USA; 3 Christopher Bond Life Sciences Center, University of Missouri, Columbia, Missouri, USA; 4 Department of Veterinary Pathobiology, University of Missouri, Columbia, Missouri, USA; 5 Division of Clinical Microbiology, Department of Laboratory Medicine, Karolinska Institutet, Stockholm, Sweden

**Keywords:** LonP1, mitochondria, ATP-dependent protease, CDDO, allosteric inhibition, mitochondrial metabolism, proteostasis, protein quality control, ADP, adenosine diphosphate, ALL, acute lymphoblastic leukemia, AML, acute myeloid leukemia, ATP, adenosine triphosphate, BAF, barrier-to-autointegration factor, CDDO, 2-cyano-3,12-dioxooleana-1,9(11)-dien-28-oic acid, CODAS, cerebral, ocular, dental auricular and skeletal, IKK-β, inhibitor of nuclear factor kappa-B kinase subunit beta, Jak2, Janus kinase 2, Keap1, Kelch-like ECH-associated protein 1 i, LXRα, liver X receptor alpha, mTOR, mechanistic/mammalian target of rapamycin, PXR, pregnane X receptor, Stat3, signal transducer and activator of transcription 3

## Abstract

The mitochondrial protein LonP1 is an ATP-dependent protease that mitigates cell stress and calibrates mitochondrial metabolism and energetics. Biallelic mutations in the *LONP1* gene are known to cause a broad spectrum of diseases, and LonP1 dysregulation is also implicated in cancer and age-related disorders. Despite the importance of LonP1 in health and disease, specific inhibitors of this protease are unknown. Here, we demonstrate that 2-cyano-3,12-dioxooleana-1,9(11)-dien-28-oic acid (CDDO) and its -methyl and -imidazole derivatives reversibly inhibit LonP1 by a noncompetitive mechanism, blocking ATP-hydrolysis and thus proteolysis. By contrast, we found that CDDO-anhydride inhibits the LonP1 ATPase competitively. Docking of CDDO derivatives in the cryo-EM structure of LonP1 shows these compounds bind a hydrophobic pocket adjacent to the ATP-binding site. The binding site of CDDO derivatives was validated by amino acid substitutions that increased LonP1 inhibition and also by a pathogenic mutation that causes cerebral, ocular, dental, auricular and skeletal (CODAS) syndrome, which ablated inhibition. CDDO failed to inhibit the ATPase activity of the purified 26S proteasome, which like LonP1 belongs to the AAA^+^ superfamily of ATPases Associated with diverse cellular Activities, suggesting that CDDO shows selectivity within this family of ATPases. Furthermore, we show that noncytotoxic concentrations of CDDO derivatives in cultured cells inhibited LonP1, but not the 26S proteasome. Taken together, these findings provide insights for future development of LonP1-specific inhibitors with chemotherapeutic potential.

Mitochondrial LonP1 is a cell-stress response protease that selectively eliminates misassembled or damaged proteins (1, 2, 3) and also degrades certain rate-limiting proteins regulating mitochondrial metabolism and energetics (4, 5, 6, 7). Growing evidence shows that the role of LonP1 in disease progression is tissue- and organ-specific and mechanistically complex, extending well beyond just protein quality control (1, 2). The mechanistic complexities mediated by LonP1 are highlighted by the distinctly different disease phenotypes associated with pathogenic variants in the nuclear *LONP1* gene (1, 2, 8). The first disease identified to be caused by biallelic *LONP1* missense mutations was CODAS syndrome, a rare developmental disorder characterized by cerebral, ocular, dental auricular and skeletal anomalies (9, 10). Other nonoverlapping *LONP1* mutations have subsequently been identified, which are distinguished either by profound neurologic dysfunction (5), congenital diaphragmatic hernia (11), mitochondrial encephalopathy (12) or classical mitochondrial DNA depletion-related symptoms (13). The mechanistic pathways derailed by LonP1 dysfunction that underlie this broad spectrum of genetic diseases remain unclear. Defective LonP1 expression and activity have also been implicated in a variety of more common diseases of the brain, heart, muscle as well as aging (14). In addition, the upregulation of LonP1 has been observed in various solid tumors and blood cancers and is postulated to be a risk factor for promoting oncogenesis (4, 6, 15, 16, 17). However, details are lacking as to the mechanisms by which increased LonP1 expression might alleviate proteotoxic, hypoxic, and oxidative stress and reprograms mitochondrial energetics and metabolism in cancer growth and how these functions can be exploited for chemotherapeutic benefit.

Our previous work showed that the protease activity of LonP1 is inhibited by the synthetic triterpenoid 2-cyano-3,12-dioxooleana-1,9(11)-dien-28-oic acid (CDDO) and its methyl derivative CDDO-Me (also known as bardoxolone-methyl) (Fig. 1*A*) (15). These compounds have been shown to promote cancer cell death (17, 18, 19), implicating LonP1 as an anticancer drug target. CDDO and its derivatives (Fig. 1*A*) have also been proposed to regulate anti-inflammatory and antioxidative stress response pathways (20, 21, 22, 23). Multiple cellular targets are directly inhibited by CDDO derivatives, such as Keap1 (24, 25), PPARγ (26, 27), IκB kinase beta (IKK-β) (28), Jak1 and Stat3 (29), mTOR (30) and tubulin (31). Over the years, CDDO derivatives have been or are being investigated in clinical trials for treating advanced solid tumors and lymphomas, pulmonary arterial hypertension (32), chronic kidney disease/diabetic kidney disease (33, 34, 35), autosomal dominant polycystic kidney disease (36) and Alport syndrome (37).

**Figure 1:**
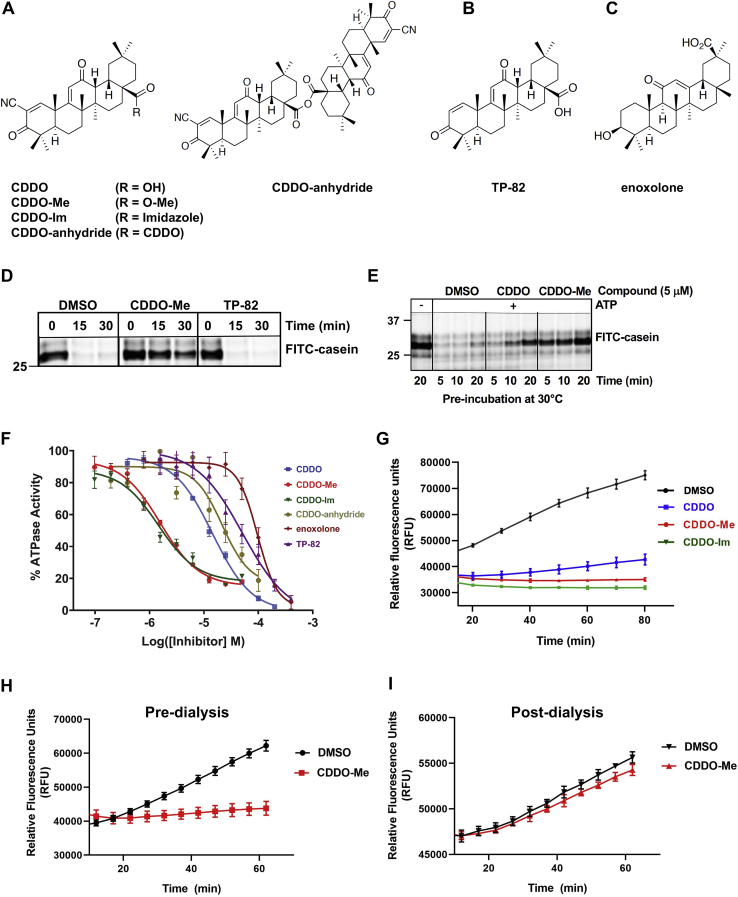
**The triterpenoids CDDO and its derivatives inhibit the protease and ATPase activities of LonP1.** Structures of (*A*) electrophilic CDDO derivatives; (*B*) TP-82, a nonelectrophilic CDDO analog that lacks the C-2 electron-withdrawing group; (*C*) enoxolone, another pentacyclic triterpenoid. *D*, CDDO and CDDO-Me inhibit LonP1-mediated degradation of FITC-casein, whereas TP-82 does not. LonP1 (1.0 μM, monomer) was preincubated (30 min, 30 °C) with or without inhibitor or DMSO vehicle control (≤1%). Reactions were initiated by adding FITC-casein (0.1 mg/ml) and ATP (4 mM) followed by incubation at 37 °C for the indicated times. Representative of N ≥ 3 independent experiments. *E*, effect of preincubation time on LonP1 inhibition by CDDO derivatives at 30 °C. Reactions were initiated by adding FITC-casein (0.1 mg/ml) and ATP (4 mM) followed by incubation at 37 °C for 30 min. *F*, CDDO derivatives inhibit the ATPase activity of LonP1. A dose–response curve is shown for each compound tested. LonP1 (400 nM, monomer) was preincubated with compound (60 min, 25 °C), after which ATP (1 mM final) was added, and reactions were incubated (60 min, 25 °C), quenched and luminescence measured using the ADP-Glo endpoint assay. The error bars indicate the SD of replicate experiments (N = 4). *G*–*I*, kinetics of LonP1 inhibition by CDDO derivatives. Reactions were carried out as in *D* and *E* and relative fluorescence units (RFU) were measured using a plate reader. *H* and *I*, reversibility of CDDO-Me inhibition of LonP1 before and after dialysis. ATP-dependent degradation of FITC-casein was performed as in *D* and *E*. *H*, before dialysis, CDDO-Me inhibition of LonP1 was determined. An aliquot (50 μl) of the reaction mixture (500 μl) containing LonP1 incubated with CDDO-Me (10 μM) or the DMSO control was removed and the kinetics of FITC-casein degradation was assayed. *I*, after dialysis, the remainder of the reaction mixture in (*H*) was transferred to a Slide-A Lyzer cassette and dialyzed overnight at 4 °C with Buffer K (50 mM Hepes KOH, pH 8.0, 150 mM NaCl, 10 mM MgOAc_2_, 20% glycerol). After dialysis, the kinetics of FITC-casein degradation was assayed as in *G*. CDDO, 2-cyano-3,12-dioxooleana-1,9(11)-dien-28-oic acid.

Here, we report the mechanism by which LonP1 is inhibited by CDDO derivatives and identify the compound binding pocket in the homohexameric LonP1 complex. Using biochemical approaches, we demonstrate that CDDO, CDDO-Me, and CDDO-Im inhibit LonP1 by a noncompetitive mechanism of inhibition by blocking ATP binding and hydrolysis, whereas CDDO-anhydride inhibits LonP1 competitively. In addition, we docked these compounds into the cryo-EM structure of human LonP1 (PDB 7NGF) (38) and validated the binding pocket by engineering amino acid substitutions of key binding pocket residues. Our results show that CDDO derivatives bind at two nonoverlapping sites within a hydrophobic pocket contiguous with a channel lined by polar residues forming salt bridges, which is adjacent to the ATP/ADP-binding site. The geometry of this binding site was scrutinized by engineered amino acid substitutions within the pockets that led to increased inhibition by CDDO and by a naturally occurring pathogenic missense LonP1 mutation that blocked inhibition. Lastly, we showed that CDDO does not inhibit the ATPase activity of purified 26S proteasome and noncytotoxic concentrations of CDDO derivatives in cultured cells blocked LonP1 but not the 26S proteasome. Taken together, these findings demonstrate that CDDO derivatives inhibit LonP1 by a new mechanism and also reveal the topological properties of an unidentified compound binding site, which can be exploited for developing chemical probes and chemotherapeutic agents to specifically target this essential cell stress response regulator.

## Results

### CDDO and its derivatives inhibit LonP1 by blocking ATP -binding and -hydrolysis

**Figure 2:**
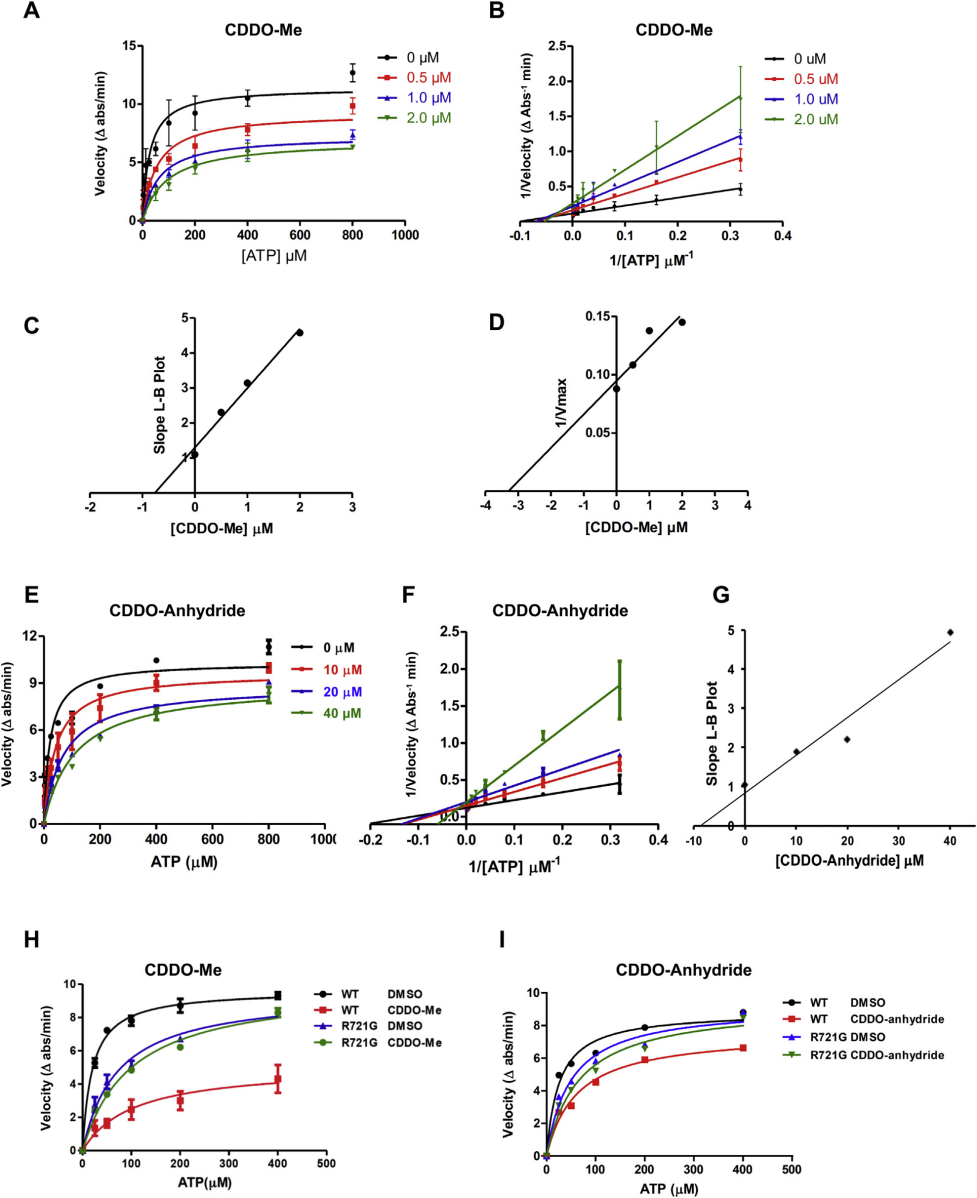
**The ATPase activity of wild-type LonP1**^**WT**^**is blocked by CDDO-Me and CDDO-anhydride by noncompetitive and competitive inhibition, respectively, whereas the CODAS mutant LonP1**^**R721G**^**ATPase is resistant to inhibition.** ATPase activities were measured using an NADH-coupled ATPase assay. Wild type and mutant LonP1 proteins (400 nM) were pre-incubated with or without compound for 30 min at room temperature. *A* and *E*, LonP1^WT^, and (*H* and *I*) comparison of LonP1^WT^ and LonP1^R721G^. ATP was titrated and its hydrolysis was measured over 5 min (mean ± S.D., N ≥ 2). *B* and *F*, double-reciprocal Lineweaver–Burk plots (mean ± S.D., N ≥ 2). *C* and *G*, determination of Ki (mean ± S.D., N ≥ 2). *D*, determination of αKi (mean ± S.D., N ≥ 2). *C*, *G* and *H*. mean values obtained from linear regression data. CDDO, 2-cyano-3,12-dioxooleana-1,9(11)-dien-28-oic acid.

While our prior work showed that CDDO and CDDO-Me inhibited protein degradation by both purified and cellular LonP1 (15), the precise mechanism of inhibition remained unknown. Here, we demonstrate that CDDO derivatives (Fig. 1*A*) inhibited not only the ATP-dependent protease activity of LonP1 as shown by degradation of fluorescently-labeled casein (FITC-casein) (Fig. 1 *D*, *E* and *G*), but also the ATPase activity as shown by endpoint assays (Fig. 1*F*) and continuous enzyme-coupled assays (Figs. 2 and S3). CDDO-Me and -Im inhibited the ATPase activity of LonP1 with greater potency than CDDO and CDDO-anhydride (Fig. 1*F*). CDDO-anhydride is a derivative in which the R group is another CDDO molecule (Fig. 1*A*). Notably, TP-82, which is identical to CDDO but lacks the electron-withdrawing nitrile moiety (Fig. 1*B*) (21, 29, 39) did not block ATP hydrolysis (Fig. 1*F*) and also failed to inhibit the degradation of FITC-casein by LonP1 (Fig. 1*D*). Similarly, a pentacyclic triterpenoid enoxolone, lacking an electron-withdrawing group, did not inhibit the LonP1 ATPase (Fig. 1*F*). These results show that the electron-withdrawing moiety of CDDO derivatives is crucial for inhibiting LonP1. By contrast, compounds such as bortezomib and MG262 that are known to inhibit LonP1’s protease activity (6, 15, 40) do not alter its ATPase activity (Fig. S1). Bortezomib and MG262 inhibit LonP1 by covalently binding to its proteolytic active site (40, 41). These compounds are high-affinity inhibitors of the 26S proteasome (42, 43), and bortezomib is a chemotherapeutic drug for treating multiple myeloma and mantle cell lymphoma (44).

We further showed that CDDO derivatives are slow-binding and reversible inhibitors of LonP1. The inhibitory effect of CDDO on ATP-dependent proteolysis was most pronounced after ≥20 min preincubation at 30 °C (Fig. 1, *E* and *G*), whereas CDDO-Me and -Im inhibited LonP1 after only 5 min at 30 °C (Figs. 1, *E* and *G* and S2*B*). The reversibility of inhibition by CDDO derivatives was demonstrated by preincubating LonP1 for 60 min at 30 °C with CDDO-Me with a high concentration of compound (10 μM) to completely block the degradation of FITC-casein (Fig. 1*H*), followed by dialyzing the reaction mixture at 4 °C for 24 h, which restored proteolytic activity (Fig. 1*I*). Collectively, these results support the notion that CDDO derivatives reversibly block the ATPase activity of LonP1, and that inhibition occurs by a mechanism independent of peptide bond hydrolysis.

### The LonP1 ATPase is inhibited noncompetitively by CDDO, CDDO-Me, and CDDO-Im, whereas CDDO-anhydride inhibits LonP1 competitively

To determine the mechanism of inhibition, enzyme kinetic assays were performed using an NADH coupled ATPase assay (Fig. 2) after preincubating LonP1 with compound or DMSO for 30 min at 25 °C. The noncompetitive inhibition of LonP1 by CDDO derivatives was demonstrated by determining the apparent Ki (αKi), apparent V_max_, Ki, and αKi values (Fig. 2, *A*–*D* for CDDO-Me and Fig. S3 for CDDO and CDDO-Im). Noncompetitive inhibition was readily apparent in the Lineweaver–Burk plots that produced plots with the same X-intercept (Figs. 2*B* and S3, *B* and *F*) but different Y-intercepts. CDDO, CDDO-Me, and CDDO-Im had α-values of 5.4, 4.3, and 1.1, respectively (Table 1), which indicated a higher affinity of the inhibitor for the free enzyme. We noted that the apparent Km increased with increasing inhibitor concentration, whereas the apparent Vmax decreased, suggesting that increasing substrate concentration was unable to overcome inhibition (Fig. 2*A*). Collectively, these results demonstrate a noncompetitive mechanism by which CDDO and its methyl and imidazole derivatives inhibit the ATPase and protease activities of LonP1 and a competitive mechanism of inhibition by CDDO-anhydride (Fig. S4).

**Table 1:** CDDO, CDDO-Me, and CDDO-Im inhibit the LonP1 ATPase by a noncompetitive mechanism, whereas CDDO-anhydride inhibits by a competitive mechanism

Compound	Modality	Ki (μM)	αKi (μM)	α	IC_50_ (μM)	N
CDDO	noncompetitive	2.6 ± 0.5	14.0 ± 2.0	5.4	13 ± 6	6
CDDO-Me	noncompetitive	0.8 ± 0.1	3.3 ± 1.0	4.3	1.9 ± 0.3	4
CDDO-Im	noncompetitive	1.9 ± 0.7	2.0 ± 0.1	1.1	2.0 ± 0.8	3
CDDO-anhydride	competitive	9.5 ± 1.4	N/A	N/A	19.4 ± 3.3	2

Ki, αKi, and α were determined from continuous ATPase assays. For competitive inhibition, α Ki, and α are not applicable (N/A). Error values for Ki and αKi represent the uncertainty about the x-intercept associated with linear regression. IC_50_ values were determined from end-point ATPase assays. They were derived from the best-fit dose–response curves and are reported as the mean ± S.D. of independent experiments (N).

The K_i_ values for CDDO, CDDO-Me, and CDDO-Im inhibition of LonP1 were 2.6 ± 0.5, 0.8 ± 0.1, and 1.9 ± 0.7 μM, respectively (Table 1 and Figs. 2*C* and S3, *D* and *H*). The Ki was determined by Lineweaver–Burk plots. The inhibitory dissociation constants for E and ES complex (Ki and αKi, respectively) were independent of the substrate concentration by contrast to IC_50_ values (Table 1). Ki was determined by plotting the reciprocal of V_max_ against the inhibitor concentration and the X-intercept provided an absolute values of αK_i_. The αK_i_ values for CDDO, CDDO-Me, and CDDO-Im were 14 ± 2, 3.3 ±1.0, and 2.0 ± 0.1 μM, respectively (Table 1 and Figs. 2*D* and S3, *C* and *G*). The αKi values of these compounds were determined by plotting the reciprocal of V_max_ against the inhibitor concentration and fitting the data by linear regression. The α values for CDDO and CDDO-Me were 5.4 and 4.3, respectively (Table 1), whereas the α for CDDO-Im was 1.1, and hence the Ki and αKi are nearly equal representing a unique case where the Ki and IC_50_ are predicted to be equal and independent of the substrate concentration.

### Identification of a unique binding pocket for CDDO derivatives adjacent to the LonP1 ATP-binding site

**Figure 3:**
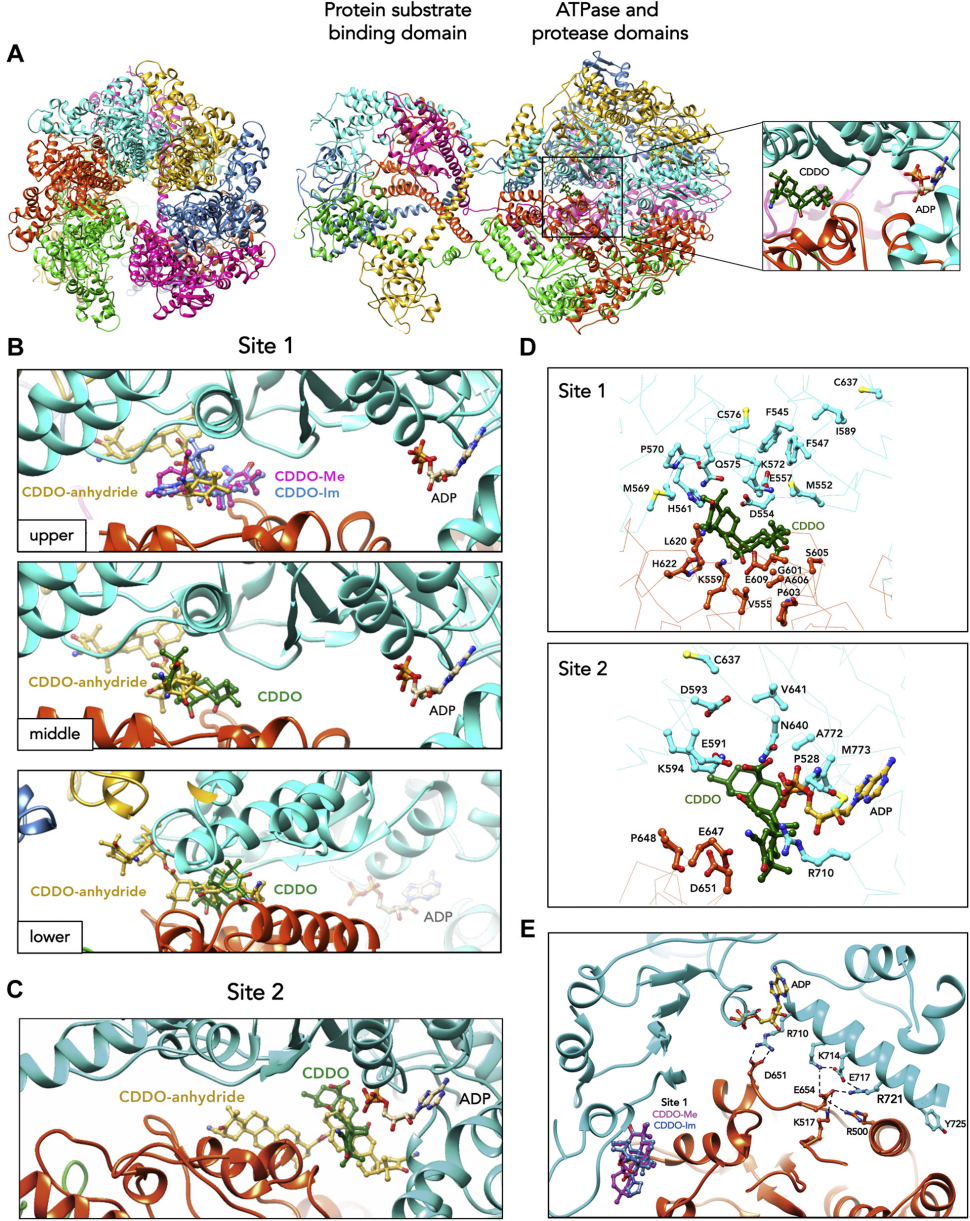
**Docking of CDDO derivatives at an allosteric binding pocket adjacent to the ATP/ADP binding site of LonP1.***A*, cryo-EM structure of LonP1. *Left*, C-terminal view clearly showing the six homohexameric subunits of the LonP1 complex. *Middle*, 90° anticlockwise rotation of the C-terminal view showing the amino-terminal protein substrate binding domain, the ATPase and protease domains. *Right*, inset showing ATP-bound between two adjacent subunits and CDDO docked at Site 1. *B* and *C*, Site 1 and Site 2, respectively are nonoverlapping pockets that bind CDDO derivatives. *B*, *upper panel*, Site 1 is more distal to the ATP/ADP binding site than Site 2. At this site, CDDO-Me, CDDO-Im and CDDO interact with two adjacent subunits (*cyan* and *orange*). *Middle panel*, CDDO and CDDO-anhydride also bind Site 1 and are shown separately for clarity. *Lower panel*, shows from a different angle that CDDO-anhydride interacts with 3 adjacent subunits at Site 1 (*cyan*, *orange*, *gold*) by contrast to CDDO, CDDO-Me and CDDO-Im, which interact with two subunits. *C*, only CDDO and CDDO-anhydride are bound at Site 2, which is more proximal to the ATP/ADP binding site than Site 1. *D*, locations of amino acids C576, C637 and F547 at the CDDO binding Site 1 and 2. Shown are the side chains of residues on two adjacent subunits (*cyan* and *orange*) of LonP1, which are affected by the amino acid substitutions. *E*, a channel is formed by a relay of inter- and intra-subunit salt bridges leading to the binding sites for CDDO derivatives and ATP. Amino acid residue R721 forms a salt-bridge with E517 of the same subunit at the opening of the channel. The missense mutation substituting R721 to glycine (R721G) encoded by the homozygous *LONP1* Amish allele of CODAS syndrome is expected to collapse this channel leading to the CDDO binding sites. CDDO, 2-cyano-3,12-dioxooleana-1,9(11)-dien-28-oic acid.

Cryo-EM structures of human LonP1 consisting of the ATPase and protease domains have been reported (45) [Protein Data Bank (PDB) entries 7SKL, 7SKM, 7KRZ]. More recently, cryo-EM structures of near full-length LonP1 have been solved. These structures include the amino-terminal substrate-binding domain, as well as the ATPase and protease domains through which an endogenous protein substrate is threaded (38) (PDB entries 7NGF, 7NFY, 7NGP, 7NGY, 7NG5, 7NG4, 7NGC, 7NGQ, 7NGL). While any of these nearly complete structures could be used to identify the binding pocket and to dock CDDO derivatives, we selected PDB entry 7NGF for our analysis, which contained bound ATP/ADP and the polypeptide substrate. The C-terminal end view of this LonP1 complex, clearly shows a homohexameric form of the protease (Fig. 3*A*, left). A 90° anticlockwise rotation of the LonP1 complex with bound ADP/ATP shows the protein substrate-binding domain together with the ATPase and protease domains of LonP1 (Fig. 3*A*, middle); the threaded polypeptide chain has been removed for clarity. The inset shows CDDO docked between two adjacent subunits, and ATP also bound between these subunits (Fig. 3*A*, right). Our docking results showed that CDDO derivatives were bound at two nonoverlapping sites within a hydrophobic channel at the interface between subunits adjacent to the ATP/ADP-binding site. We designated these sites as Site 1, which is distal to bound ATP/ADP (Fig. 3*B*) (at 20 Å away), and Site 2, which is more proximal (at ∼4 Å away) to the ATP/ADP-binding site (Fig. 3*C*). CDDO-Me, CDDO-Im, CDDO, and CDDO-anhydride could be docked at Site 1, consisting of amino acids from two adjacent LonP1 subunits (Fig. 3*B* upper and middle panels, cyan and orange subunits). By contrast CDDO-anhydride interacted with three adjacent LonP1 subunits (Fig. 3*B*, lower panel, cyan, orange, and gold). Only CDDO and CDDO-anhydride could be docked at Site 2 (Fig. 3*C*). As CDDO-Me and CDDO-Im are the more potent inhibitors of LonP1 (Fig. 1, *F* and *G* and Table 1), these docking results suggest that Site 1 is the principal allosteric binding pocket for inhibition by these compounds.

The different binding pocket geometries of CDDO-anhydride as compared with CDDO, CDDO-Me, and CDDO-Im were in line with biochemical data demonstrating that CDDO-anhydride had a distinctly different mechanism of LonP1 inhibition from the other CDDO derivatives. Lineweaver–Burk plots showed that CDDO-anhydride blocked the LonP1 ATPase by a competitive mechanism of inhibition as the lines nested on the Y-intercept (Fig. 2*F*), which is in contrast with the other CDDO derivatives that showed noncompetitive inhibition (Figs. 2*B* and S3, *B* and *F*). There was significant increase in apparent K_m_ for ATP with minimal decrease in the V_max_. Additionally, LonP1 inhibition by CDDO-anhydride was overcome by increased ATP concentration, further supporting a competitive mechanism of inhibition. In the presence of CDDO-anhydride, the ATPase activity of LonP1 had a Ki of 9.5 ± 1.4 μM (Fig. 2, *E*–*G* and Table 1), which was ∼3.5 fold higher than the Ki of other CDDO derivatives. Together, these findings support the proposition that the binding of CDDO-anhydride to LonP1 alters the geometry of the ADP/ATP-binding pocket or that the compound-binding pocket overlaps the ATP/ADP-binding site.

### LonP1 inhibition by CDDO is enhanced by mutagenesis of cysteine residues near the hydrophobic CDDO-binding pocket

The proposed mechanism by which CDDO derivatives inhibit target proteins is through the formation of Michael adducts between these electrophilic compounds and nucleophilic groups within the protein (*e.g.*, free thiol groups on cysteine residues) (25, 46). For example, specific cysteine residues have been identified in Keap1 (24, 25), IKK-β (28), Jak2, and Stat3 (29), which are required for inhibition by CDDO derivatives. Thus, we tested if a similar mechanism of adduct formation might exist for LonP1 inhibition.

Two cysteine residues at positions 576 (C576) and 637 (C637) are located near a hydrophobic cluster adjacent to the CDDO binding Sites 1 and 2 (Fig. 3*D*). Conservative amino acid substitutions were engineered by replacing both cysteine residues with the hydrophobic residue valine (C576V and C637V) or with the polar residue serine (C576S and C637S). In addition, phenylalanine 547 (F547), also positioned within the same hydrophobic cluster (Fig. 3*D*, Site 1) was replaced by alanine (F547A). Intriguingly, C576V, C637V, C637S, and F547A did not ablate inhibition by CDDO; instead, these mutations increased inhibition as demonstrated by reduced IC_50_ values (Table 2). Single C576V, C637V, and F547A substitutions reduced IC_50_ values from 2 to 5-fold, whereas double mutations of both cysteine and phenylalanine significantly reduced the IC_50_ values further by 7 to 8-fold (Table 2). Compared with wild-type LonP1 with an IC_50_ value of 14 μM, the double LonP1 mutants C576V-F547A and C637V-F547A had IC_50_ values for CDDO, which were 1.8 and 1.9 μM, respectively (Table 2). Control experiments showed that the mutant LonP1 proteins with amino acid substitutions showed ATP-stimulated peptidase activities (Fig. S5, *A* and *B*). Collectively, these data suggest that C576, C637, and F547 residues are critical for the geometry of the CDDO-binding pocket, and that C576 and C637 do not participate in the formation of adducts with CDDO derivatives thereby leading to LonP1 inhibition.

**Table 2:** Amino acid substitutions of phenylalanine 547, cysteines 637 and 576 near the CDDO-binding pocket increase compound inhibition of LonP1

LonP1	CDDO IC_50_ (μM)	Fold increase in inhibition compared with WT
WT	14.0 ± 2	-
F547A	5.9 ± 0.6 ∗∗	2.4
C576V	5.2 ± 1.5 ∗∗	2.7
C637V	3.7^a^	3.8
F547A/C576V	1.8 ± 0.8 ∗∗∗	7.8
F547A/C637V	1.9 ± 0.3 ∗∗∗	7.4
C576S	11.9^a^	1.2
C637S	3.0^a^	4.7

IC_50_ values were determined from end-point ATPase assays. They were derived from the best-fit dose–response curves and are reported as the mean ± S.D. of independent experiments (N ≥ 2) except for ^a^ where N = 1. (∗∗*p* < 0.01, ∗∗∗*p* < 0.001 by Tukey–Kramer multiple comparison test).

To gain insight into how amino acid substitutions at C576, C637, and F547 might introduce structural changes in LonP1, we conducted mutant modeling using Schrödinger Suite “Prime” (Schrödinger LLC, NY) followed by quantum mechanics/molecular mechanics (QM/MM) minimization using “Jaguar.” The solvent accessibility surface area (SASA) of C576 and C637 is 45 and 38 Å^2^, respectively. As an example, results showed that the C576V substitution altered the side chain orientation of compound-binding pocket residues, increasing the number of interactions with CDDO (Fig. S6). The analysis of binding Site 1 showed that C576V most likely increases the hydrophobicity of the hydrophobic cluster formed by F545, F547, I589, and the carbon chain of K572 (Fig. 3*D*, Site 1, cyan carbons). Increased hydrophobicity would reduce the flexibility of the compound binding pocket (Site 1), thereby stabilizing the interaction of binding site residues with CDDO (Fig. 3*D*, Site 1). Several residues on the same side of C637 that interact with CDDO derivatives include M569, H561, Q575, K572, D554, E557, and M552 (Fig. 3*D*, Site 1, cyan). Several residues from the neighboring subunit and opposite to C637 interact with CDDO, which include L620, H622, K559, E609, V555, P603, A606, G601, S605 (Fig. 3*D*, Site 1, orange). Amino acids F547 and F545 are buried and do not directly interact with CDDO derivatives, but they are within van der Waals interacting distance from K572, E557, and M552, which directly interact with CDDO (Fig. 3*D*, Site 1, cyan). Therefore, we suggest that the mutation F547A reorients K572 closer to CDDO. By contrast to the C637V mutation, the impact of the C637S substitution is not clear from the modeled structures. It is possible that the serine substitution at C637 interacts with the backbone N-H or C=O moieties, thereby reducing the flexibility of the binding pocket leading to stronger interactions between binding site residues and CDDO derivatives (Fig. 3*D*, Site 1, cyan), thus increasing compound affinity and inhibition. Our interpretation is speculative, and we acknowledge that the mutant-dependent decrease in IC_50_ values may be explained by other mechanisms as well. Interestingly at Site 2, E591 and K594, which are at the Walker Motif B of the ATP-binding site, QM/MM showed interaction with CDDO (Fig. 3*D*, Site 2, cyan). The conserved E591 and K594 residues in homologs of LonP1 from bacteria to humans are essential for activating a water molecule leading to the nucleophilic attack on the γ-phosphate of ATP (47). Additional residues that interact with CDDO include D593, V641, N640, A772, M773, P528, and R710 from one subunit (Fig. 3*D*, Site 2, cyan) and P648, E647, and D651 from the adjacent subunit (Fig. 3*D*, Site 2, orange). Although, it is possible that CDDO forms adducts with cysteine residues elsewhere in LonP1 leading to inhibition as has been shown for other CDDO target proteins, this appears unlikely as there are no other cysteine residues located in proximity of the compound-binding pockets.

### Resistance to CDDO derivatives is conferred by the pathogenic mutation LonP1^R721G^

We were curious to know whether a naturally occurring pathogenic mutation in LonP1 might alter inhibition by CDDO derivatives. Biallelic mutations in the chromosomal gene encoding human LonP1 cause CODAS syndrome (9, 10). We examined the purified recombinant LonP1 mutant with the homozygous Amish CODAS mutation in which arginine 721 was replaced by glycine (LonP1^R721G^) and showed that it retained partial ATPase activity with a K_m_ for ATP of 71.70 ± 3.14 μM and a V_max_ of 9.51, as compared with wild-type LonP1^WT^ with a Km of 19.81 ± 0.72 μM and a V_max_ of 9.66 (Fig. 2, *H* and *I*). We have previously shown that the LonP1^R721G^ mutant also retains ATP-dependent protease activity albeit reduced as compared with LonP1^WT^ (Fig. S7) (9). Interestingly, LonP1^R721G^ showed resistance to CDDO-Me (20 μM), as compared with LonP1^WT^ (Fig. 2*H*). Similarly, LonP1^R721G^ also showed resistance to CDDO-anhydride (20 μM) (Fig. 2*I*). These data suggest that pathogenic mutation within the *LONP1* gene, as well as nonpathogenic polymorphisms, can influence compound sensitivity and may be informative and potentially exploited in compound design and chemotherapeutic applications.

Molecular modeling using the same cryo-EM structure of LonP1 provided some insights into the mechanism by which the CODAS mutation R721G ablated inhibition by CDDO derivatives (Fig. 3*E*). R721 is at the interface of two neighboring subunits near the CDDO-binding pocket leading to a relay of salt bridges. The interface has multiple polar interactions appearing to form a flexible channel lined by a number of salt bridges on adjacent subunits (Fig. 3*E*, cyan and orange), which may permit the entry of CDDO derivatives to their respective binding pockets. R721 on one subunit (cyan) forms a salt bridge with E654 from the adjacent subunit (orange), which also forms a salt bridge with R500 of the same subunit (orange). E654 (orange) also forms a salt bridge with K517 (orange), which forms a salt bridge with E717 (cyan) that forms a salt bridge with K714 (cyan). Another salt bridge between R710 (cyan) and D651 (orange) is proximal to the CDDO binding at Site 1. It is possible that the missense mutation R721G likely disrupts this network of salt bridges, altering channel geometry such that the new topology at the subunit interface hinders binding of CDDO derivatives at Site 1. Additionally, substitution of R721 by the smaller glycine side chain is expected to generate significant a solvent accessible region for the increased dissociation rate of compounds from the binding pocket, reducing compound binding affinity and therefore decreased efficacy of inhibition by CDDO derivatives.

### CDDO derivatives selectively inhibit LonP1 but not the 26S proteasome

**Figure 4:**
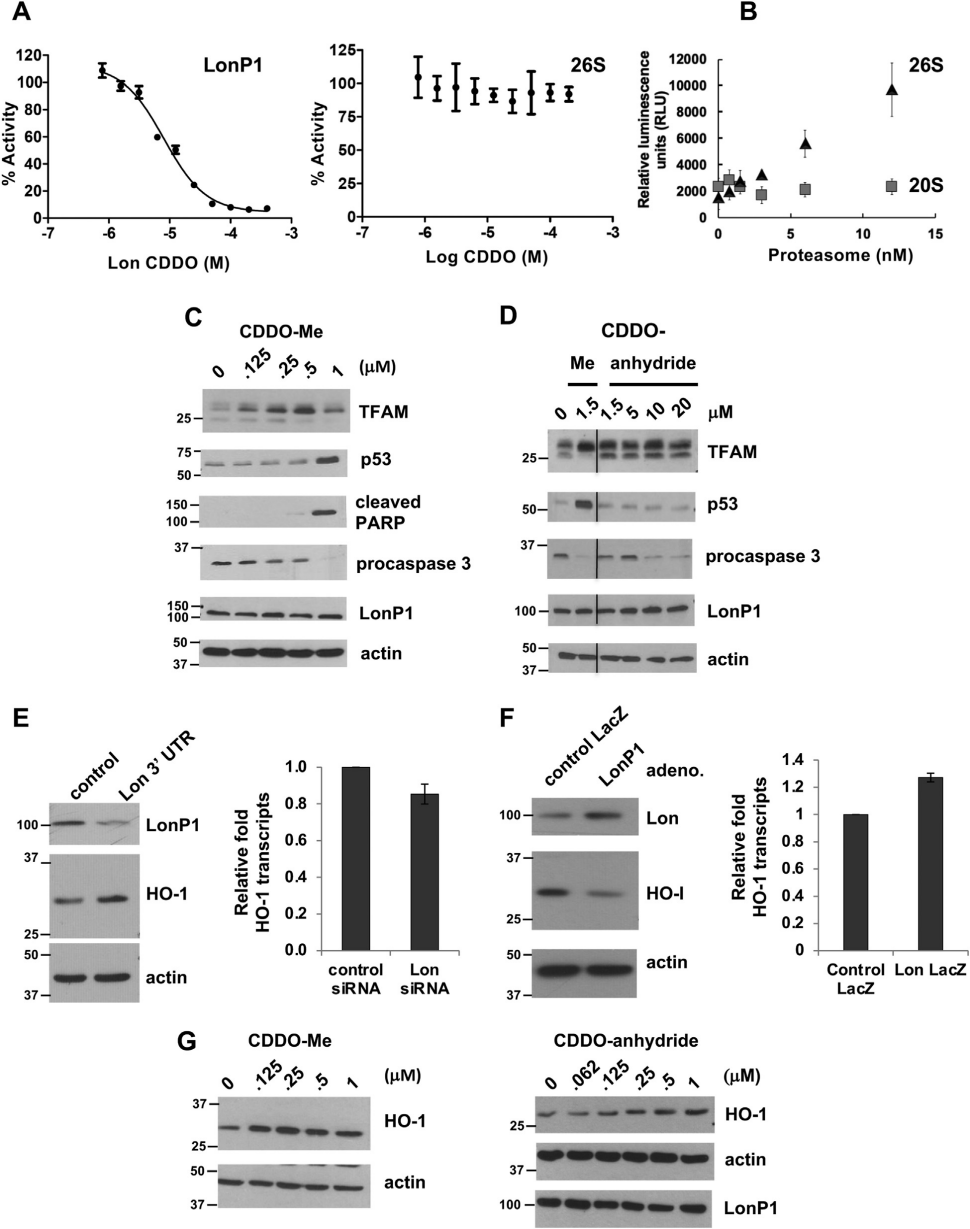
**CDDO-Me and CDDO-anhydride selectively inhibit LonP1 but not the 26S proteasome.***A*, CDDO inhibited the ATPase activity purified LonP1, however, it failed to inhibit the ATPase activity of the 26S proteasome. *B*, control experiments show that in the absence of CDDO, the 26S proteasome hydrolyzed ATP in a concentration-dependent manner whereas the 20S particle lacking the 19S particle had no ATPase activity. *C* and *D*, HeLa ρ^0^ cells were treated with CDDO-Me or -anhydride for 7 h at the concentrations shown, after which cells were harvested, proteins extracted and then immunoblotted. β-actin was used as a loading control. *E* and *F*, levels of HO-1 protein and transcripts were examined in HEK293 T cells either- (*E*)*,* knocked down for LonP1 using siRNA targeting the 3′UTR of the *LONP1* transcript treated for 4 days; or (*F*)*,* overexpressing human LonP1 using an adenovirus delivery system for 48 h. For immunoblotting, β-actin was used as loading control, whereas for qRT-PCR, *GAPDH* was used to normalize HO-1 transcript levels. *G*, HeLa ρ^0^ cells were titrated with CDDO -Me or -anhydride for 7 h, and cells were harvested, protein extracted, and immunoblotted for HO-1, LonP1, or β-actin. CDDO, 2-cyano-3,12-dioxooleana-1,9(11)-dien-28-oic acid.

LonP1 belongs to the AAA^+^ superfamily of ATPases Associated with diverse cellular Activities, which mediate various cellular processes such as DNA replication, recombination, chromatin remodeling, ribosomal RNA processing, membrane fusion as well as ATP-dependent proteolysis (48). The 26S proteasome consists of the 20S proteolytic core particle, which mediates peptide bond hydrolysis and the 19S regulatory particle containing ATPases belonging to the AAA^+^ family (49). Although CDDO inhibited the ATPase activity of purified LonP1, it failed to inhibit ATP hydrolysis by the 26S proteasome (Fig. 4*A*). Control experiments demonstrated that in the absence of CDDO, the 26S proteasome hydrolyzed ATP in a concentration-dependent manner, whereas the 20S particle lacking the 19S component did not have ATPase activity (Fig. 4*B*). We sought to test the apparent selectivity of CDDO derivatives for LonP1 but not the 26S in cultured cells. There are no known proteins that are constitutively degraded by LonP1 under baseline conditions, which would permit us to assay protease inhibition by CDDO derivatives. Therefore, we employed experimentally derived HeLa cells lacking mitochondrial DNA (mtDNA) (referred to as HeLa ρ^0^ cells) (50), in which LonP1 constitutively degrades mitochondrial transcription factor A (TFAM) (6). When TFAM fails to bind mtDNA (*e.g.*, when mtDNA is absent), it is rapidly degraded by LonP1, thus providing a readout for protease activity (6). An advantage of using this system is that HeLa cells express the human papillomavirus (HPV) E6 protein, which promotes constitutive degradation of p53 by the 26S proteasome. Thus, the effects of CDDO derivatives on LonP1 and the 26S proteasome can be examined in parallel.

LonP1-mediated proteolysis of TFAM was inhibited by CDDO-Me at 0.125 to 0.5 μM, which did not block 26S proteasome-dependent degradation of p53 (Fig. 4*C*). CDDO-Me at 1 μM led to increased protein levels of p53; however, ≥1 μM CDDO-Me was cytotoxic, inducing apoptosis as demonstrated by the increased levels of cleaved PARP and decreased levels of procaspase 3, which are indicative of caspase 3 activation (Fig. 4*C*). No change in the protein levels of LonP1 or actin was observed. Consistent with our previous observation, CDDO-Me primarily stabilized the full-length TFAM precursor protein (6) (Fig. 4*C*). Like CDDO-Me, CDDO-anhydride (1.5–20 μM) selectively and effectively inhibited TFAM degradation by LonP1 (Fig. 4*D*), stabilizing the TFAM precursor as well as the mature form albeit to a lesser extent (Fig. 4*D*). The TFAM precursor may accumulate because of inefficient processing of the full-length polypeptide resulting from changes in mitochondrial membrane fluidity induced by CDDO derivatives as previously suggested (51). CDDO-anhydride even at 20 μM did not block the 26S proteasome-mediated degradation of p53 (Fig. 4*D*). No change in procaspase 3 levels was observed up to 5 μM. However, concentrations of CDDO-Me >10 μM were cytotoxic, leading to decreased levels of procaspase 3, indicating its cleavage and the activation of apoptosis (Fig. 4*D*). CDDO-anhydride had no effect on the protein levels of LonP1 or actin.

To examine the effects of CDDO-anhydride and CDDO-Me in cells that had an intact mitochondrial genome, we examined another potential protein substrate of LonP1, heme oxygenase 1 (HO-1). Published work suggested that LonP1 constitutively degrades HO-1, which catalyzes the degradation of heme within mitochondria (52). To demonstrate that HO-1 is a LonP1-substrate, we knocked down or overexpressed LonP1 in HEK293T cells. LonP1 knockdown stabilized HO-1 protein, whereas LonP1 overexpression decreased HO-1 protein (Fig. 4, *E* and *F*). In both cases, there was no significant change in HO-1 transcript levels, thus under these conditions, LonP1 protein levels did not affect HO-1 transcription (Fig. 4, *E* and *F*). This finding was also observed in the HeLa ρ^0^ cells, which showed that CDDO-Me stabilized HO-1 at ≥0.125 μM, and CDDO-anhydride stabilized HO-1 at ≥0.25 μM (Fig. 4*G*). Taken together, these data support the conclusion that noncytotoxic concentrations CDDO-Me and CDDO-anhydride selectively inhibit LonP1 but not the 26S proteasome without induction of apoptosis and suggest that CDDO derivatives can show target selectivity within the AAA^+^ family of ATPases.

## Discussion

New opportunities and approaches are needed for the development of specific, high-affinity inhibitors and activators of human LonP1, which is an essential mitochondrial protease in human health and disease. The development of allosteric inhibitors and activators of LonP1 will be invaluable in elucidating its mechanistic and functional complexities and holds promise for chemotherapeutic benefit in treating cancers and age-associated disorders such as atherosclerosis and neurodegeneration. The overexpression of LonP1 has been observed in numerous solid tumors and blood cancers and is postulated to be a risk factor for promoting oncogenesis (4, 6, 15, 16, 17, 53). Emerging evidence suggests that inhibiting LonP1 or other quality control proteins in mitochondria (54, 55) and endoplasmic reticulum (56) of cancer cells or immunosuppressor cells (57) is a potential strategy for disabling oncogenic progression. The endoplasmic reticulum (ER) and mitochondrial unfolded protein response pathways (UPR^ER^ and UPR^mt^, respectively) have been postulated to impart an advantage to cancer cells, supporting cell survival, proliferation and evasion of immunosurveillance, and drug resistance (57, 58, 59, 60), by mitigating hostile conditions within the tumor microenvironment such as nutrient and oxygen deprivation, oxidative stress, and high metabolic demand.

Previous studies have identified inhibitors of LonP1 that bind its proteolytic active site (40, 41, 61). We showed that bortezomib, which is used clinically to treat multiple myeloma and mantle cell lymphoma by potently blocking the 20S proteasome with an IC_50_ of 2.3 nM, also inhibits LonP1 with an IC_50_ of 17 nM for LonP1 (6). Subsequently, X-ray crystallography and cryo-EM have demonstrated that bortezomib binds the proteolytic active site of LonP1 and Lon-like proteins (40, 41, 45). Whether LonP1 inhibition contributes to the therapeutic benefit of bortezomib or is instead an off-target detriment is unknown. Obtusilactone A and (-)-sesamin have also been shown to inhibit LonP1 with IC_50_ values of 34.1 μM and 19.9 μM, respectively (61). These compounds were proposed to bind the proteolytic active site as determined by homology modeling and molecular docking (61). Obtusilactone A and sesamin are reported to have multiple cellular targets. The direct interaction between Obtusilactone A with barrier-to-autointegration factor (BAF) has been demonstrated (62), and sesamin has been found to bind to Annexin A1, liver X receptor alpha (LXRα) and pregnane X receptor (PXR) (63, 64).

In this study, we demonstrate that CDDO, CDDO-Me, and -Im are allosteric noncompetitive inhibitors of the LonP1 ATPase, which directly block ATP binding and hydrolysis and hence they also inhibit ATP-dependent proteolysis. Allosteric inhibitors offer potential advantages in the development of protein-specific compounds as they do not bind primary orthosteric active sites, which are often highly conserved. Instead, they bind at remote sites that modulate active site conformation. As the geometry of allosteric-binding sites is frequently unique or with limited representation in cellular protein structures, this increases the potential for target specificity and selectivity. In addition, noncompetitive inhibitors bind not only to the free enzyme but also to the enzyme-substrate complex, thus inhibition is unaffected by fluctuating substrate concentrations. By contrast, CDDO-anhydride is a competitive inhibitor, which like CDDO-Me also inhibits LonP1 in cells (Fig. 4, *D* and *G*).

We have shown that CDDO derivatives inhibit LonP1 but not the 26S proteasome, suggesting that these compounds have specificity within the superfamily of AAA^+^ ATPases. It is possible that CDDO derivatives may interfere with other AAA^+^ proteins such as the mitochondrial matrix ClpXP protease. Recent work has identified small-molecule inhibitors and activators of ClpXP (65, 66, 67), which have shown chemotherapeutic potential in treating malignancies associated with acute myeloid leukemia (AML), acute lymphoblastic leukemia (ALL), breast cancer (54). Thus, experiments are being performed to test whether CDDO derivatives inhibit ClpXP as well as LonP1.

We anticipate that allosteric noncompetitive inhibitors of LonP1 and competitive inhibitors binding to Sites 1 and 2 as identified in this study can be employed alone or combined with proteolytic active site inhibitors for the efficacious inhibition of LonP1 and other AAA^+^ proteases. As LonP1 can function as an ATP-dependent chaperone independent of its protease activity (68, 69, 70), compounds that inhibit ATP binding and hydrolysis will effectively block both chaperone and protease activities. Furthermore, the identification of allosteric ATPase inhibitors of LonP1 suggests the likelihood of developing allosteric activators to promote its roles as an energy-dependent protease and chaperone.

## Experimental procedures

### Reagents

CDDO and CDDO-Methyl (CDDO-Me) were purchased from Cayman Chemical, CDDO-Imidazole (CDDO-Im) was purchased from Tocris. CDDO-anhydride (20) and TP-82 (71) were synthesized as reported. Other inhibitors in this study were purchased commercially: MG262 (Boston Biochem), bortezomib (LC Labs) and enoxolone (Cayman Chemical). Antibodies recognizing the following antigens were employed in this study: LonP1 (Proteintech, cat. 15440-1-AP), p53 (Calbiochem, cat. 0P43, lot # D00086815), cleaved PARP (Asp214) (BD Biosciences, cat. 552596), procaspase-3 monoclonal antibody (Transduction Laboratories, cat. C31720), actin (Santa Cruz, cat. sc-1615, lot # F0408), heme oxygenase 1 (Santa Cruz, cat. sc-136960, lot # B1516), TFAM kindly provided by Daniel Bogenhagen (Stony Brook University). Human 26S and 20S proteasome purified from human embryonic kidney cells (HEK293) were purchased commercially (Boston Biochem Inc.-R&D Systems, cat. E-365, lot # 35730210 and cat. E-360, lot # 16918510, respectively).

### LonP1 purification

Human mitochondrial LonP1 and mutants lacking the predicted mitochondrial targeting sequence were fused to an N-terminal hexa-histidine affinity tag, expressed in Rosetta 2 *E. Coli*, and purified using a nickel agarose column as previously described (72) and protein concentration and buffer exchange carried out using Amicon Ultra centrifugal filtration. LonP1 mutant constructs were obtained from the PCR-based QuikChange Site-Directed Mutagenesis Kit.

### FITC-casein protease assay

Human LonP1 (1.0 μM monomer) was preincubated with CDDO derivatives, TP-82, enoxolone (5 μM) or DMSO vehicle control (1%) in buffer (150 mM NaCl, 50 mM Hepes-KOH pH 8.0, 10 mM MgCl_2_, 0.1 mg/ml BSA) for 30 min at 30 °C. Reactions were initiated by the addition of FITC-casein (0.1 mg/ml) and ATP (4.0 mM) and incubated at 37 °C. The kinetics of FITC-casein degradation was measured by the increase in relative fluorescence units (RFU) at 490–525 nm wavelength at 37 °C using a Spectramax or Biotek Synergy plate reader. For endpoint assays, aliquots of reactions incubated at 37 °C were removed at indicated time points and terminated by adding 5X reducing sample buffer (RSB). The decrease of intact FITC-casein was determined by SDS-PAGE followed by visualization using FluorChem or Chemi-Doc systems.

### Reversibility of CDDO-Me inhibition

Reactions (500 μl) containing LonP1 (1.0 μM monomer) in buffer (150 mM NaCl, 50 mM Hepes-KOH pH 8.0, 10 mM MgCl_2_) were preincubated with CDDO-Me (10 μM) or DMSO (1%) for 60 min at 30 °C. After this period, respective aliquots (50 μl) were removed and assayed for FITC-casein degradation to confirm inhibition by CDDO-Me. The remaining 450 μl reaction was transferred to a Slide-A-Lyzer (100 kDa MWCO) and dialyzed against 500 ml Buffer K (50 mM Hepes KOH, pH 8.0, 150 mM NaCl, 10 mM MgOAc_2_, 20% glycerol) for 24 h at 4 °C. After this period, the protein concentration was measured to determine protein recovery and the protease activity of LonP1, which had been preincubated with and without CDDO-Me was measured using the FITC-casein degradation assay.

### Endpoint ATPase assay

ADP-Glo (Promega) assays were performed according to the manufacturer's protocols. Purified wild-type or mutant LonP1 (400 nM monomer), the 26S proteasome (3 nM), or no enzyme controls were preincubated in 96-well plates (60 min, 25 °C) with ten different concentrations of CDDO and its derivatives, TP-82, enoxolone (5 μM) or DMSO vehicle control in buffer (40 mM Tris-HCl (pH 7.5), 20 mM MgCl_2_, 0.1 mg/ml BSA and 5% DMSO). The 26S and 20S proteasome were also assayed at 0, 0.75, 1.5, 3, 6, and 12 nM in the absence of CDDO derivatives. UltraPure ATP (1 mM final) was added, and reactions (50 μl final) were incubated for 60 min at 25 °C, then 5 μl was transferred in quadruplicate to a 384-well plate. Assay reagents were used to quench further ATPase activity and generate luminescence signals proportional to the concentration of ADP formed. Background (no enzyme control) luminescence values were subtracted, and resultant values were reported as a percentage of the no drug control (100%). Data were fit to four-parameter dose–response curves using GraphPad Prism. The error bars represent the standard deviation (SD) of four replicate reactions from at least three independent experiments.

### Continuous NADH-coupled ATPase assay

ATPase reactions (200 μl total) were performed in 96-well plates at room temperature. LonP1 (400 nM monomer) was preincubated with DMSO (1–2%) vehicle control or CDDO derivatives in buffer (50 mM HEPES-KOH pH 7.5, 5 mM magnesium acetate, 75 mM potassium acetate) for 30 to 45 min at room temperature or 30 °C. The reactions were initiated by the addition of phosphoenolpyruvate (PEP, 3 mM), NADH (300 μM), pyruvate kinase and lactate dehydrogenase (PK-LDH) (12–20 U/ml), and ATP concentrations as indicated. The change in NADH absorbance at 340 nm was monitored in Spectramax plate reader for 5 min to obtain reaction velocities. Data were fit to the Michaelis–Menten curve using GraphPad Prism 5, and the error bars represent the standard deviation (SD) of four replicate reactions from at least three independent experiments.

### Identification of binding sites for CDDO and its derivatives

The cryo-EM structure of the LonP1 complex composed of nearly full-length subunits (PDB NGF) consisting of the amino-terminal protein substrate binding domain, the ATPase and protease domains, was used for identifying compound-binding pockets and docking of the CDDO derivatives. Three independent molecular modeling programs were used: (i) Q-siteFinder, (ii) SiteMap (Schrödinger Suite, NY), and (iii) SiteID (Certara, Tripos Associates, St Louis, MO). Prior to subjecting the structure of LonP1 to the SiteMap program, Protein Preparation Wizard (Schrödinger Suite, NY) was used to add hydrogen atoms, partial charges, protonation states, and optimization of H-atoms. The final structure was subjected to restrained minimization (1000 iterations) by ‘Impact’ program of Schrödinger Suite with OPLS_2005 force field. The other pocket finding software Q-SiteFinder and SiteID did not require addition of hydrogen bonds and partial charges; hence, the unaltered cryo-EM structure of LonP1 was used for CDDO-binding pocket analyses. In all three programs, the bound ATP and ADP as reported in the cryo-EM structure were taken into account. Potential CDDO-binding sites predicted by the three programs were visually inspected for their size and shape complementarity with the CDDO derivatives. Two pockets were predicted by all three programs (SiteMap, Q-siteFinder and SiteID) for the docking of CDDO, CDDO-Me, CDDO-Im, and CDDO-anhydride near the ATP/ADP-binding site.

### Docking of CDDO derivatives

The docking of CDDO derivatives was conducted as follows. First, the molecular models of CDDO, CDDO-Me, CDDO-Im, and CDDO-anhydride were generated from “structure data files” (sdf) obtained from PubChem. The 3D structures of these compounds as well as their tautomeric forms were generated by using LigPrep, a ligand preparation tool of Schrödinger Suite. These structures were then docked into the selected binding sites using the “induced-fit docking” utility of the software “Glide” (Schrödinger Suite). The “Induced Fit Docking” workflow allowed the optimization of side chains in the binding pocket to filter the compounds for best binding energy. The top two poses exhibiting best binding energy were selected for structural analyses. All docking was done in the presence of ATP and ADP as reported in the cryo-EM structure. The charges on ADP/ATP were calculated by semiempirical quantum mechanical method PM3 (73, 74).

### Generation of the molecular models of LonP1 in complex with CDDO derivatives

The specific amino acid substitutions in LonP1 were generated by “Prime” utility of Schrödinger Suite. The hexameric cryo-EM structure of LonP1 was subjected to limited minimization (1000 steps) by “Impact” using the OPLS_2005 force field followed by molecular dynamics simulation. First, we applied the Quantum Mechanical/Molecular Mechanical (QM/MM) protocol using Q-site (Schrödinger Inc. NY) to calculate the partial charges on CDDO and its derivatives and ADP molecule, and on side chains within 6 Ǻ from both ligands. These charges were subsequently used to perform molecular dynamics (MD) simulations for 10,000,000 steps with 50 femtosecond step size. All the atoms more than 20 Å away from ADP and CDDO derivatives were constrained to their mean position in MD simulations.

### Cell culture, transfection, and drug treatment

HeLa cells devoid of mitochondrial DNA (HeLa ρ^0^) and LCL cells were generated as described previously (50). DMEM, RPMI, and fetal bovine serum were purchased from Sigma. All cells were grown with 5% CO_2_ at 37 °C. LCL cells were cultured in RPMI supplemented with 15% FBS. HeLa Rho 0 cells were cultured in high glucose DMEM (25 mM) (Sigma) supplemented with sodium pyruvate (110 mg/ml) and uridine (50 mg/ml). CDDO-Me was purchased from Cayman chemicals, and CDDO-AH was synthesized as described previously (20). HEK293 cells plated in 60 mm dishes at a confluency of 60 to 70% were transfected with Control or 3′UTR LonP1 siRNA using Lipofectamine 2000 (Invitrogen) and reduced serum OPTI MEM (Sigma) as described in the manufacturer’s protocol to knockdown LonP1, whereas adenoviral transduction was employed to overexpress LonP1. CDDO-Me and CDDO-anhydride were dissolved in DMSO and serially diluted in the respective complete medium and added to HeLa ρ^0^ cells and were treated for 7 or 24 h.

### Immunoblotting

Cells were harvested, centrifuged, and washed with PBS and proteins were extracted by adding the lysis buffer (50 mM Tris, [pH 7.5], 300 mM NaCl, and 0.5% Triton X-100) containing 2X Halt phosphatase and protease cocktail inhibitor (Thermo Scientific) for 20 min on ice. After centrifugation at 14,000 rpm for 15 min at 4 °C, the supernatant was collected and estimated for protein by Bradford’s method. In total, 30 to 40 μg of protein was separated by SDS-PAGE and immunoblotted with respective antibodies.

### Quantitative PCR analysis

RNA was isolated using RNAeasy kit (Qiagen) as described in the manufacturer’s protocol. In total, 500 ng of RNA was converted to cDNA using cDNA conversion kit (Applied Biosystems); 50 ng cDNA was used to estimate the relative quantification of HO-I transcripts after normalizing with GAPDH transcripts. The relative quantification (RQ) was calculated using CFX 96 software (Bio Rad) by ΔΔCt method and expressed as RQ ± standard error mean (SEM).

## Data availability

All data are included within the manuscript.

## Supporting information

This article contains supporting information

## Conflict of interest

The authors declare that they have no conflict of interest with the contents of this article.
