# Supplementary material for: Inhibition of mitochondrial LonP1 protease by allosteric blockade of ATP binding and hydrolysis via CDDO and its derivatives
Source: J Biol Chem. 2022 Feb 11;298(3):101719. doi: 10.1016/j.jbc.2022.101719 (PMC8921294; doi:10.1016/j.jbc.2022.101719)
Supplement: Supplemental Figures S1–S7 [file mmc1.pdf]

Inhibition of mitochondrial LonP1 protease by allosteric blockade of ATP -binding and -hydrolysis via CDDO and its derivatives

Jae Lee,[1,7] Ashutosh K. Pandey,[1,6,7] Sundararajan Venkatesh,[1] Jayapalraja Thilagavathi,[1]

Tadashi Honda,[2] Kamal Singh,[3,4,5] and Carolyn K. Suzuki[1,8]

[1]Department of Microbiology, Biochemistry and Molecular Genetics,

Rutgers - New Jersey Medical School, Newark, NJ 07103, USA

[2]Department of Chemistry and Institution of Chemical Biology & Drug Discovery,

Stony Brook University, Stony Brook, NY 11794, USA

[3]Christopher Bond Life Sciences Center, University of Missouri, Columbia, MO 65212, USA

[4]Department of Veterinary Pathobiology, University of Missouri, Columbia, MO 65212, USA

[5]Department of Laboratory Medicine, Division of Clinical Microbiology,

Karolinska Institutet, Stockholm, SE-141 86, Sweden

[6]Current location: Department of Pharmacology, Physiology and Neuroscience,

Rutgers- New Jersey Medical School, Newark, NJ 07101, USA

[7]Co-first authors

*Correspondence: suzukick@njms.rutgers.edu (C.K.S.), singhka@missouri.edu (K.S.)

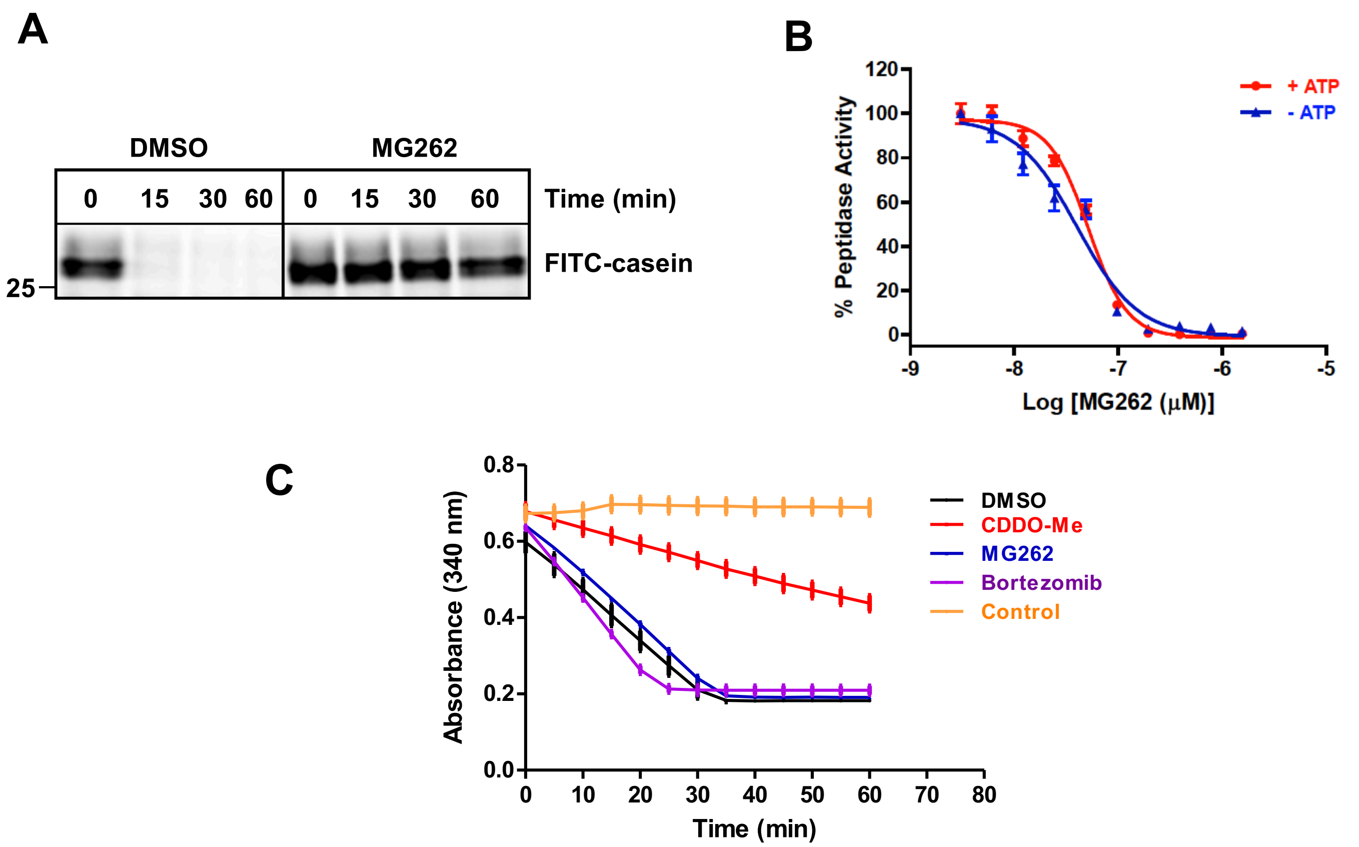

Figure S1. The proteolytic active site inhibitors bortezomib and MG262 do not inhibit the ATPase activity of LonP1. (A) MG262 inhibits the ATP-dependent protease activity of LonP1. LonP1 (1.0 µM, monomer) was pre-incubated (30 min, 30°C) with or without inhibitor or DMSO vehicle control (≤1%). Reactions were initiated by adding FITC-casein (0.1 mg/ml) and ATP (4 mM) followed by incubation at 37°C. At indicated time points, aliquots were removed, and the reaction terminated by adding reducing sample buffer. The decrease of intact FITC-casein was determined by SDS-PAGE followed by visualization using FluorChem or Chemi-Doc systems. Representative of N ≥ 3 independent experiments. **(B) MG262 and bortezomib inhibit the peptidase activity of LonP1 in the presence or absence of ATP.** Purified Lon (800 nM monomer) was incubated in reaction buffer (150 mM NaCl, 50 mM Hepes-KOH pH 8.0, 10 mM $MgCl_2$) with MG262 at the concentrations shown and the fluorescent dipeptide substrate rhodamine 110, bis-(CBZ-L-alanyl-L-alanine amide (RhoAA,6 µM), with or without ATP (2 mM), at 37°C for 3 hours. Fluorescence was measured at excitation/emission of 485/535 nm using a Perkin Elmer Victor3 V. The relative fluorescence units (RFUs) of the background (no enzyme control) were subtracted, and the resultant values were normalized to percent activity of the no inhibitor reactions. Data were fit to 4-parameter dose-response curves using GraphPad Prism 5, and the error bars represent the standard deviation (SD) of 4 replicate reactions. **(C) Neither MG262 nor bortezomib inhibit the ATPase activity of LonP1.** LonP1 (400 nM monomer) was pre-incubated for 30 min at 25°C with or without DMSO or the compounds as shown (10 µM). ATP (50 µM final) was added, and LonP1 ATPase activity was measured using a continuous NADH-coupled ATPase assay in which the absorbance at 340 nM was measured for 1 hr at 25°C.

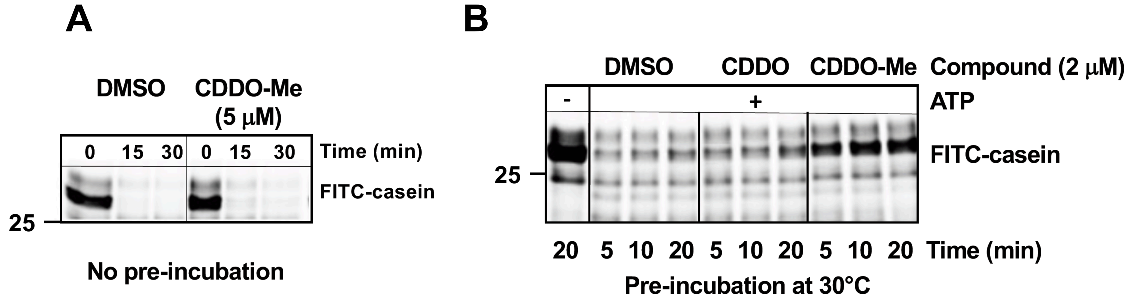

Figure S2. Pre-incubation of CDDO derivatives with LonP1 promotes inhibition of ATP-dependent proteolysis. (A) No pre-incubation with CDDO-Me. LonP1 (1.0 μM, monomer) in reaction buffer with ATP (4 mM) containing CDDO-Me or DMSO control and FITC-casein (0.1 mg/ml) were incubated at 37°C. At indicated time points, aliquots were removed, and the reaction terminated by adding reducing sample buffer. The decrease of intact FITC-casein was determined by SDS-PAGE followed by visualization using FluorChem or Chemi-Doc systems. **(B)** LonP1 (1.0 μM, monomer) was pre-incubated with inhibitor (2 μM) or DMSO vehicle control (1%) at 30°C for the times as shown. Reactions were initiated by adding FITC-casein (0.1 mg/ml) and ATP (4 mM) followed by incubation for 30 min at 37°C. Reactions were terminated by adding reducing sample buffer. The decrease of intact FITC-casein was determined by SDS-PAGE followed by visualization using FluorChem or Chemi-Doc systems.

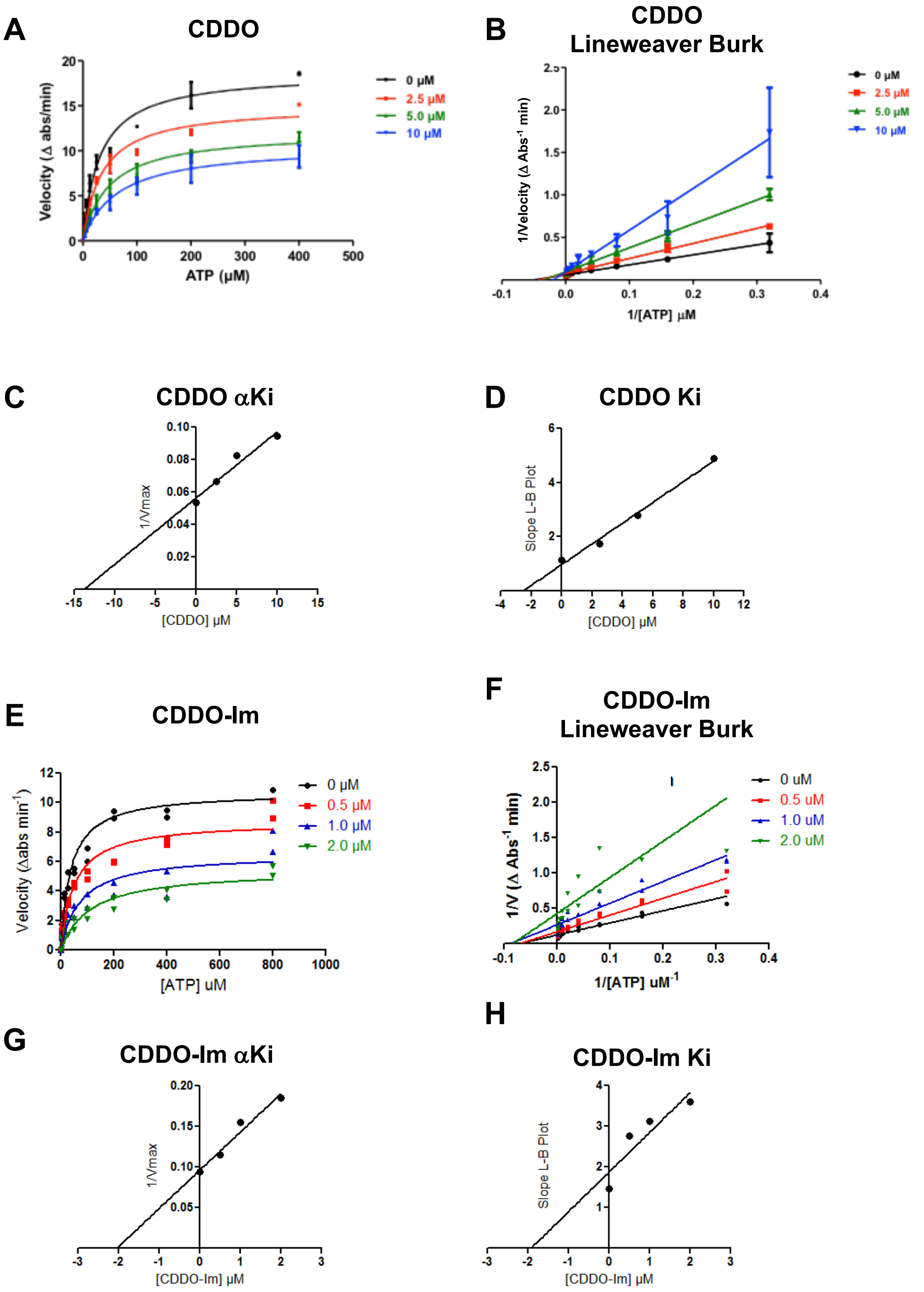

Figure S3. CDDO and CDDO-Im inhibit the LonP1 ATPase by a non-competitive mechanism. LonP1 ATPase activities were measured using an NADH-coupled ATPase assay. LonP1 (400 nM) was pre-incubated with or without compound for 30 min at room temperature. (**A and E**) Saturation curves. ATP was titrated and hydrolysis was measured over 5 min (mean ± S.D., N ≥ 2). **(B and F)** Double-reciprocal Lineweaver-Burk plots (mean ± S.D., N ≥ 2). **(C and G)** Determination of αKi (mean ± S.D., N ≥ 2). **(D and H)** Determination of Ki (mean ± S.D., N ≥ 2). (**C, D, G, H**). Mean values obtained from linear regression data.

A. Reversible non-competitive inhibition of LonP1 by CDDO, CDDO-Me and CDDO-Im

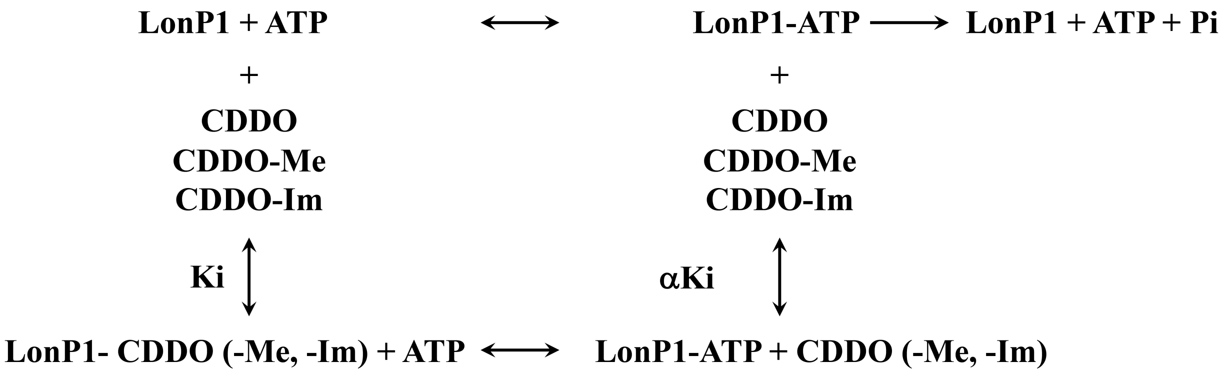

B. Competitive inhibition of LonP1 by CDDO-anhydride

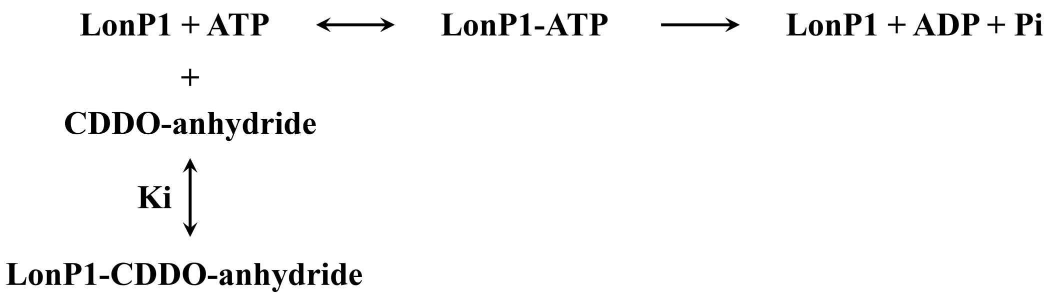

Figure S4. Mechanisms of LonP1 inhibition by CDDO derivatives. (A) Reversible non-competitive inhibition of LonP1 by CDDO, CDDO-Me, and CDDO-Im. The inhibitor binds with significant although not necessarily equal affinity to both the free enzyme (LonP1) and enzyme-substrate (LonP1-ATP) with the respective equilibrium dissociation constants represented by Ki and αKi. **(B)** Reversible competitive inhibition of LonP1 by CDDO-anhydride. The inhibitor binds primarily to the free enzyme with the equilibrium dissociation constant represented as Ki.

A

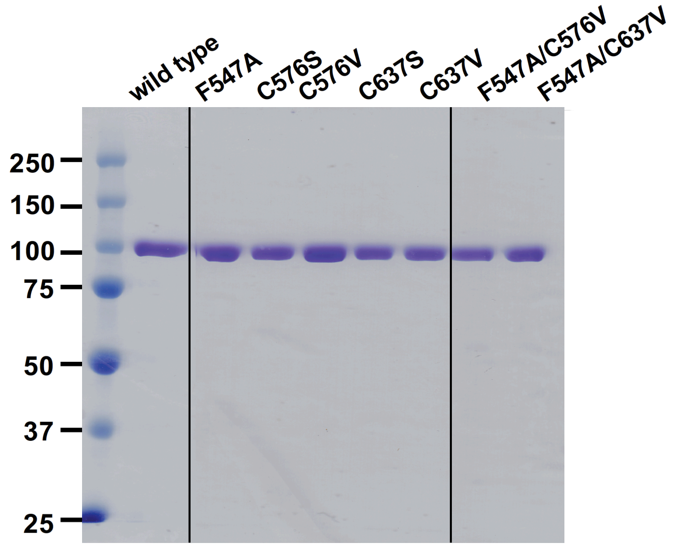

B

ATP-stimulated peptidase activity
of LonP1 wild type and CDDO binding site mutants

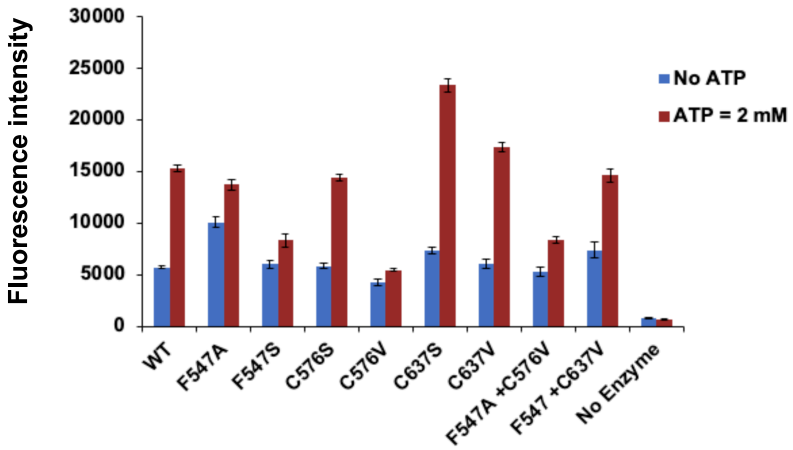

Figure S5. Purified LonP1 wild type and CDDO binding site mutants and their respective ATP-stimulated peptidase activities. (A) Purified wild type and amino acid substitution mutants of LonP1 (2 µg/lane) were run on 10% SDS-PAGE and visualized by Coomassie Brilliant Blue staining. The proteins shown were run on the same gel and the black lines indicate splice borders where irrelevant protein lanes were removed. **(B)** LonP1 wild type and mutant proteins (800 nM monomer) were incubated with the fluorescent dipeptide substrate rhodamine 110, bis-(CBZ-L-alanyl-L-alanine amide (RhoAA,6 µM), in reaction buffer (150 mM NaCl, 50 mM Hepes-KOH pH 8.0, 10 mM $MgCl_2$), with and without ATP (2 mM) at 37°C for 3 hr. Fluorescence was measured at excitation/emission of 485/535 nm using a Perkin Elmer Victor3 V. Each reaction was measured in triplicate wells of a 96 well plate. The error bars represent the standard deviation (SD) from at least 3 independent experiments.

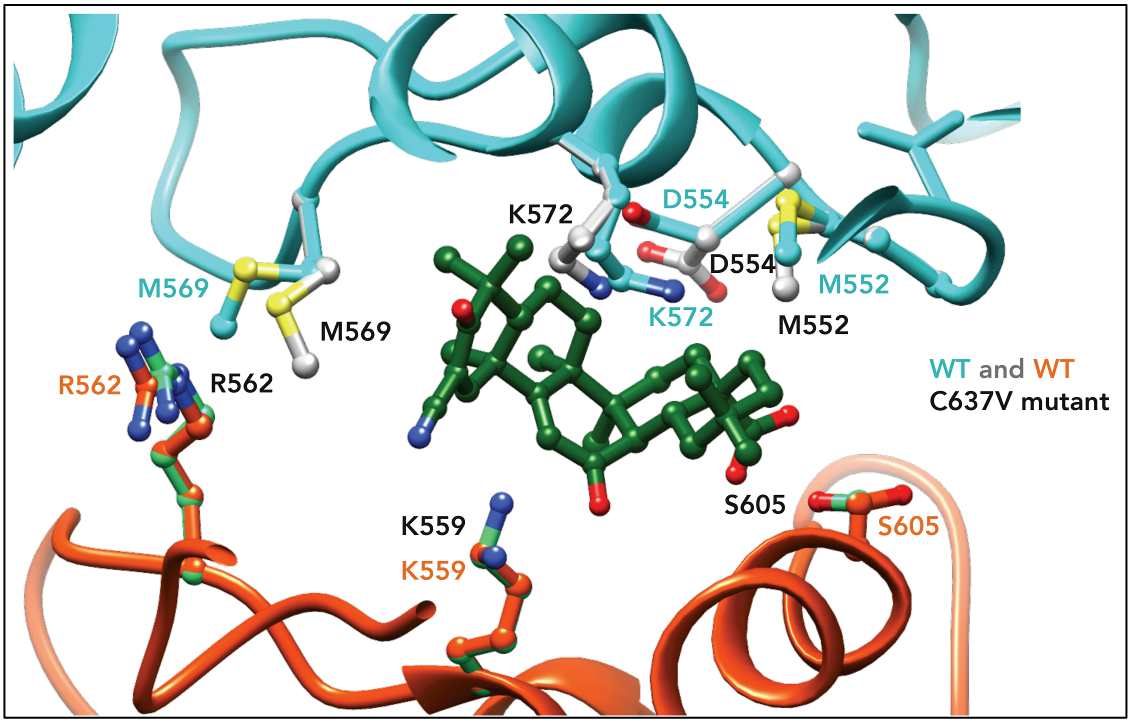

Figure S6. LonP1 mutation C637V alters the orientation of amino acid sidechains at the binding pocket increasing interactions with CDDO. To determine the effect of C637V mutation, a Quantum Mechanics/Molecular Mechanics (QM/MM) computation was conducted using Q-site program of Schrödinger Suite. For this purpose, the sidechains within 3 Å of CDDO were treated with ab initio quantum chemical method 6-31G*, whereas rest of the molecule was treated with MM. The charges on CDDO (ball-and-stick) and its derivatives were computed using quantum chemical method 6-31G*. The sidechains (ball-and-stick) conformations of two wild-type LonP1 subunits (WT) are shown in cyan and orange carbons, whereas the sidechain orientations after QM/MM minimization of the C637V mutant are shown in gray carbons in one subunit and light green carbons. Other atoms are shown as by atom type- blue (nitrogen), yellow (sulfur) and red (oxygen). The figure clearly shows that the mutation C637V changes the conformation site residues such that they orient closer to CDDO.

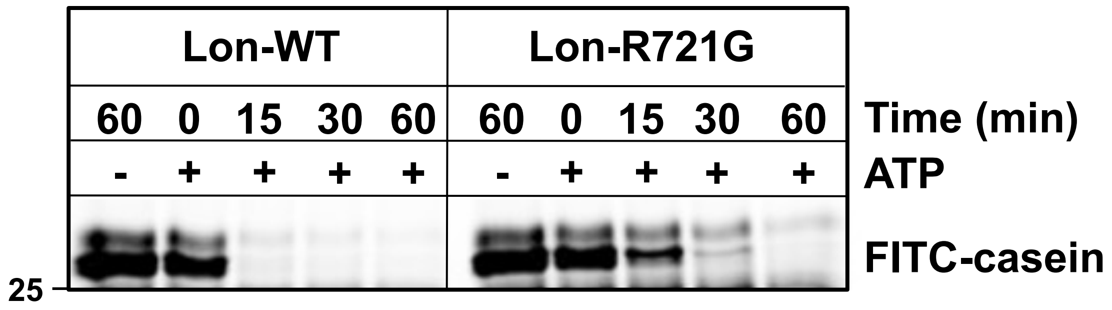

Figure S7. Lon-R721G has ATP-dependent protease activity albeit reduced as compared to wild type Lon. A master mix was prepared containing wild type LonP1 and CODAS mutant Lon^{R721G} (1μM) in buffer (50 mM HEPES KOH, pH 8.0, 150 mM NaCl and 10 mM $MgCl_2$,0.1 mg/ml BSA) with protein substrate FITC-casein (0.1 mg/ml) in the absence or presence of ATP (4 mM) at 37°C. At each time point equal aliquots were withdrawn and the reaction terminated by mixing with reducing sample buffer, and then run on a 10% SDS-PAGE. FITC-casein was imaged by phosphorimaging.
